# The effectiveness of motivational interviewing on the oral health of leukemic children and oral health care knowledge, attitude and practice of their mothers: a hospital-based intervention

**DOI:** 10.1186/s12887-023-04078-y

**Published:** 2023-05-24

**Authors:** Niloofar Falahinia, Samaneh Razeghi, Ahmad Reza Shamshiri, Manijeh Firoozi, Simin Zahra Mohebbi

**Affiliations:** 1 School of Dentistry, Tehran University of Medical Sciences, Tehran, Iran; 2 Research Center for Caries Prevention, Dentistry Research Institute, Tehran University of Medical Sciences, Tehran, Iran; 3 Community Oral Health Department, School of Dentistry, Tehran University of Medical Sciences, Tehran, Iran; 4 Cancer Research Center, Shahid Beheshti University of Medical Sciences, Tehran, Iran; 5 Department of Psychology, Faculty of Psychology and Educational Sciences, University of Tehran, Tehran, Iran

**Keywords:** Motivational interviewing, Oral health, Leukemia

## Abstract

**Background:**

Some studies suggest a higher effectiveness of motivational interviewing compared to common oral health instruction in healthy individuals. As regards to higher prevalence of dental diseases like early childhood caries, oral mucositis, and gingivitis are reported for leukemic children, the present study aims to compare the effectiveness of educating mothers through MI with the common instruction (CI) for the oral health of children with leukemia under six years old.

**Method:**

This quasi-experimental study was designed in Tehran University of Medical Sciences, School of Dentistry and conducted on 61 mothers with leukemic children under age six hospitalized in Mahak Hospital and Rehabilitation Complex which is a Pediatric Cancer Research and Hospital Center, in 2021. Mother and child pairs were allocated to MI or CI (using pamphlets) groups. Data was collected using a questionnaire of mothers’ knowledge, attitude, motivation, and practice concerning oral health care in leukemic children. The children underwent clinical examination to assess plaque index before and three months after the intervention. The data were analyzed using SPSS version 25 (IBM, Armonk, NY, USA) by ANCOVA test.

**Results:**

The preschoolers mean ages were 4.23 ± 1.41 and 4.32 ± 1.33 (ranged from 2 to 6 years old) in the MI and CI group, respectively. There were 16 girls (53.3%) and 14 boys (46.7%) in the MI group, and 15 girls (48.4%) and 16 boys (51.6%) in the CI group. Significant differences were observed in the amount of plaque index between the MI group and the CI group (0.20 ± 0.04, p-value < 0.001). A significant increase was observed in the mean of changes in scores of knowledge, attitude, motivation, mother’s practice concerning child’s oral health, mother’s practice concerning personal oral health in the MI group (p-value < 0.001).

**Conclusions:**

Considering that instruction using MI showed to be effective in improving oral health adherence in mothers and reducing plaque in children with Leukemia, it may be recommended as a promising method to promote the oral health of such susceptible children in places that they are constantly present for treatment.

**Trial registration:**

The study was registered in the Iranian Registry of Clinical Trials (IRCT) on 11.03.2021 (code: IRCT20131102015238N5).

**Supplementary Information:**

The online version contains supplementary material available at 10.1186/s12887-023-04078-y.

## Introduction

Over recent decades, cancer has been posed as one of the most serious health problems throughout the world [1]. Leukemia is the seventh most common cancer among Iranian population [2] and the most common cancer in Iranian children, also throughout the world. According to the national cancer registry in Iran in 2020, the updated Age-Standardized Rates of those suffering from leukemia is 5.3 per 100,000 people [3]. In addition, according to a 2019 report on cancer in Iran, the Age-Standardized Death Rate per 100,000 of leukemia accounted for 1.71 in children under 15 years old [4]. Moreover, the rate of improvement and survival of this cancer in children is 86%, and the therapies provided for it are chemotherapy, radiotherapy, immunotherapy, bone marrow surgery and transplantation [5]. The most common oral manifestations connected with anticancer therapies are mucositis, xerostomia, infection, salivary gland dysfunction, dysgeusia, and pain [1]. These complications can cause severe discomfort that intervenes with proper nutrition and may set back completion of therapies [6]. Also, parents probably tend to feed children high-carbohydrate and sugary food due to emotional conditions children with cancer suffer from [7]. This may increase their involvement with caries which is named as Early childhood caries (ECC) referring to one or more decayed, lost, or filled primary teeth in children aged 71 months or younger [8]. Higher prevalence of dental diseases like ECC and gingivitis are reported for leukemic children when compared to systemically healthy children [9].

ECC is both a dental and a socio-behavioral problem, affecting children in many societies. Additionally, it is considered one of the most common chronic diseases in children, particularly in developing countries [10, 11]. This disease can be related to behavioral patterns, lifestyle, low socioeconomic conditions, social rejection, and sociocultural differences in oral health beliefs and practices [12]. This type of caries is one of the most common reasons children visit dental clinic and hospitals, and they often need general anesthesia for treatment [13]. Furthermore, it can cause pain, swelling, early loss of primary teeth at a very young age, malocclusion, and other problems, all of which lead to the poor pronunciation of words, chewing and esthetics, while bringing about serious consequences for a child’s health and growth [14]. In young ages brushing should be done by the caregiver and not the child and if it is not the case, it will lead to increasing the risk of developing such caries [15]. Therefore, considering the high cost and importance of care, oral health counselling should be incorporated health settings where preschool children are taking their treatment [13].

To prevent tooth caries and reduce the consequences of treating cancer, it is necessary to educate parents and children to follow proper oral health behaviors [7, 16]. Mothers play an important role not only in facilitating oral hygiene in very young children, but also in transmitting health-related habits to children, therefore, their tooth brushing has been linked to their children’s oral cleaning frequency [17]. However, prevailing health education focuses on information giving and direct advice has been established to be ineffective in achievement sustained behavioral changes [18]. Studies suggested that instruction and education about plaque control were not effective in the long term [19]. Hence, one of the methods of changing behaviors is using the counseling technique called “Motivational Interviewing”, which was initially written about in 1983 [20]. It is a client-centered, directional counseling approach that is more focused and goal-directed than nondirective counseling. In order to encourage behavior change, Motivational Interviewing (MI) relies on identifying and mobilizing the client’s intrinsic values and goals [21]. It has been discovered that it increases change discourse and commitment while reducing resistance to therapy [22]. Therefore, from the standpoint of MI, oral health promotion essentially entails building a “patients’ eye view” and ensuring that they are aware that they are not being assessed and found wanting. It involves comprehending a patient’s viewpoint and then afterward supporting them to settle on the decisions that they value most [23]. MI is suggested to be an effective strategy to change life style and long-term maintenance of change [24, 25].

This method includes five-steps: The first stage is engaging, which means encouraging patients and reaching an agreement based on an honest relationship between doctors and patients. The second stage is focusing, which means cooperation between doctors and patients to determine the purpose of the behavior change. The third stage is evoking, which means patients’ awareness of their intrinsic motivations to change and the formation of their ideas of change. In the fourth stage, known as planning, the patients find the solutions to changing behavior and develop them with the help of the doctors. In the last stage, known as review, the patients provide doctors with feedback in the review sessions [20]. The results of some studies indicate the higher effectiveness of motivational interviewing compared to common oral health instruction in healthy individuals [16, 23]. It should be mentioned that a few studies have been conducted on the effectiveness of this method compared to common methods in maintaining oral health in people with rare diseases and children with cancer, groups at high risk of ECC [26, 27]. Therefore, the present study aims to compare the effectiveness of motivational interviewing with a common method on children’s plaque index and mothers’ knowledge, attitude, motivation and practice as regard to the oral health of their leukemic children.

## Materials and methods

### Ethics approval

Participation in the study was voluntary and the mothers signed informed consent forms prior to participation in the study. Furthermore, mothers announced their consent for their children’s participation in the study. The study had no risk or harm to participants and participants had the right to withdraw from the study at any stage. The study ensured information confidentiality and privacy. This study was approved by the Institutional Research Ethics Committee of School of Dentistry, Tehran University of Medical Sciences, code: IR.TUMS.DENTISTRY.REC.1399.208. Also, the study was registered in the Iranian Registry of Clinical Trials (IRCT) on 11.03.2021 (code: IRCT20131102015238N5).

## Trial design

This is a single-blind, parallel, quasi-experimental study with the allocation ratio of 1: 1, conducted on 61 mothers with children under age six suffering from leukemia hospitalized in Mahak Hospital and Rehabilitation Complex which is a Pediatric Cancer Research and Hospital Center June to November in 2021.

### Participants, randomisation and blinding

All participants were enrolled in the study based on inclusion and exclusion criteria. The inclusion criteria for children were: being under six years of age, receiving chemotherapy, obtaining parents’ consent for their children’s participation in the study, and having at least four teeth. The exclusion criteria were: systemic problems such as diabetes, mental disability such as cerebral palsy, and children with fever and neutropenia. After coordination with Mahak Hospital and Rehabilitation Complex authorities (which is a Pediatric Cancer Research and Hospital Center). The written consent was taken from the mothers, so that they could announce their consent for their own and their children’s participation in the study. It should be mentioned that participants’ identities were kept confidential and they were not required to mention their names. One of the authors (SZM) enrolled participants, and assigned them to the Motivation Interviewing (MI) group and the Common Instruction (CI) groups. Children with leukemia were hospitalized in four oncology wards in Mahak Hospital, which were divided into two wards for MI and two other wards for CI groups. The sampling method was carried out non-randomly and based on inclusion and exclusion criteria. The statistical analyst was blind to the groups.

### Clinical oral examinations

Prior to data collection, the last author who is an experienced dentist educated the examining dentist and they were calibrated. Before, and three months after the interventions, clinical oral examination was performed on children in both groups; Dental plaque was measured based on Löe-Silness dental plaque index (PI) [28] using a disposable dental explorer and a dental mirror.

Scoring of PI was as follow:

- Score of 0: absence of dental plaque;
- Score 1: presence of plaque in gingival margin which cannot be seen with the unaided eye;
- Score 2: a moderate amount of soft deposits in the gingival margin that can be seen with the unaided eye;
- Score 3: the presence of soft materials in the gingival margin or tooth and gingival edge.

The mean plaque of all teeth for each child was sum up as well (ranging from 0 to 3).

### Interventions

Before starting the interventions, oral assessment conducted on both groups’ children by using Löe-Silness dental plaque index. The mothers in the CI group, received an educational pamphlet, designed for the purpose of the study. It was simple and according to instructional design principles using the best updated evidence and relevant guidelines, and it was provided to mothers along with a short oral explanation by a senior dentistry student (NF).

In the MI group, motivational interviewing protocol was implemented. First, the interviewer (NF) learned the five stage executive protocol of MI while being supervised by a psychological counseling professor (MF). Then the participants in the MI group were trained individually in a 45-minute session. A dental health menu was designed, including a series of healthcare recommendations. At the end of the session, this list was provided to the parent and the mother could choose any number of recommendations to implement. The chosen strategies were provided to the parents as reminding cards and a calendar to mark the number of days each task was performed on the calendar. At the end of the sessions, two follow-up sessions were conducted with the MI group by phone after two weeks and one month. The content of the follow-up sessions included solving participants’ problems and questions, providing suggestions, and encouraging and enhancing behavior change. Furthermore, the children’s plaque index was assessed over three months after the intervention, and the parents completed the relevant questionnaire again. In addition, the instructional content provided to both MI and CI groups, was the same.

### Sample size

The two independent samples t test is used to calculate the sample size of two groups. According to the results of the study of children plaque index in Iraq [29], and considering α = 0.05, β = 20%, S1 = S2 = 0.47, and the mean difference of (µ_1_ - µ_2_) = 0.4 and also 10% loss to follow-up, minimum required total sample size was calculated equal to 64. .

### The questionnaire of the study

The data collection tools were an interview administered questionnaire including demographic information (the mother’s education, self-reported family’s financial status, child’s age and gender), nineteen questions on knowledge (3-point Likert scale), six questions on attitude (5-point Likert scale), nine questions on mother’s self-reported practice concerning children’ oral health (for instance, “Who brushes the teeth of your child?”, “How often do you take your child to the dentist”, …) and five questions on self-reported practice concerning the mother’s oral health (for example, “How often do you brush your teeth”?, “Do you use fluoridated toothpaste when brushing your teeth?”, …). Two questions (How interested are you that your child does not have dental problems?, How confident are you that you can improve your child’s oral health?) were also added to assess mother’s motivation (10-points numeric survey scale); The maximum score “1” was given to the correct or desirable answers and minimum “0” was given to the incorrect or undesirable answers. The content validity and reliability of the questionnaire were evaluated and confirmed in the previous studies [28, 30]. In addition, a form about child’s oral-health status was added at the end of questionnaire (additional file 1). The content and face validity of the questionnaire was again assessed by a group of some experts of community oral health and pediatric dentistry. Grammar, wording, proper item allocation, and scaling were examined by experts. For reliability assessment, the questionnaires were given to 12 mothers, who were not participants of the main study. They were asked to answer the questions twice with a two-week interval. For all questions, the coefficient of agreement was at least 0.9.

### Statistical analysis

The study endpoints were as follows: The PI scores ranged from 0 to 3. Mother’s knowledge, attitude, motivation, mother’s practice concerning children’s oral health, and mother’s self-reported practice concerning personal oral health were standardized to 0-100. The data were analyzed using IBM SPSS version 25.0 (IBM Corp. Released 2017. IBM SPSS Statistics for Windows, Version 25.0. Armonk, NY: IBM Corp). Data were analyzed using analysis of covariance. P-value less than 0.05 was considered as statistically significant.

## Results

**Fig. 1:**
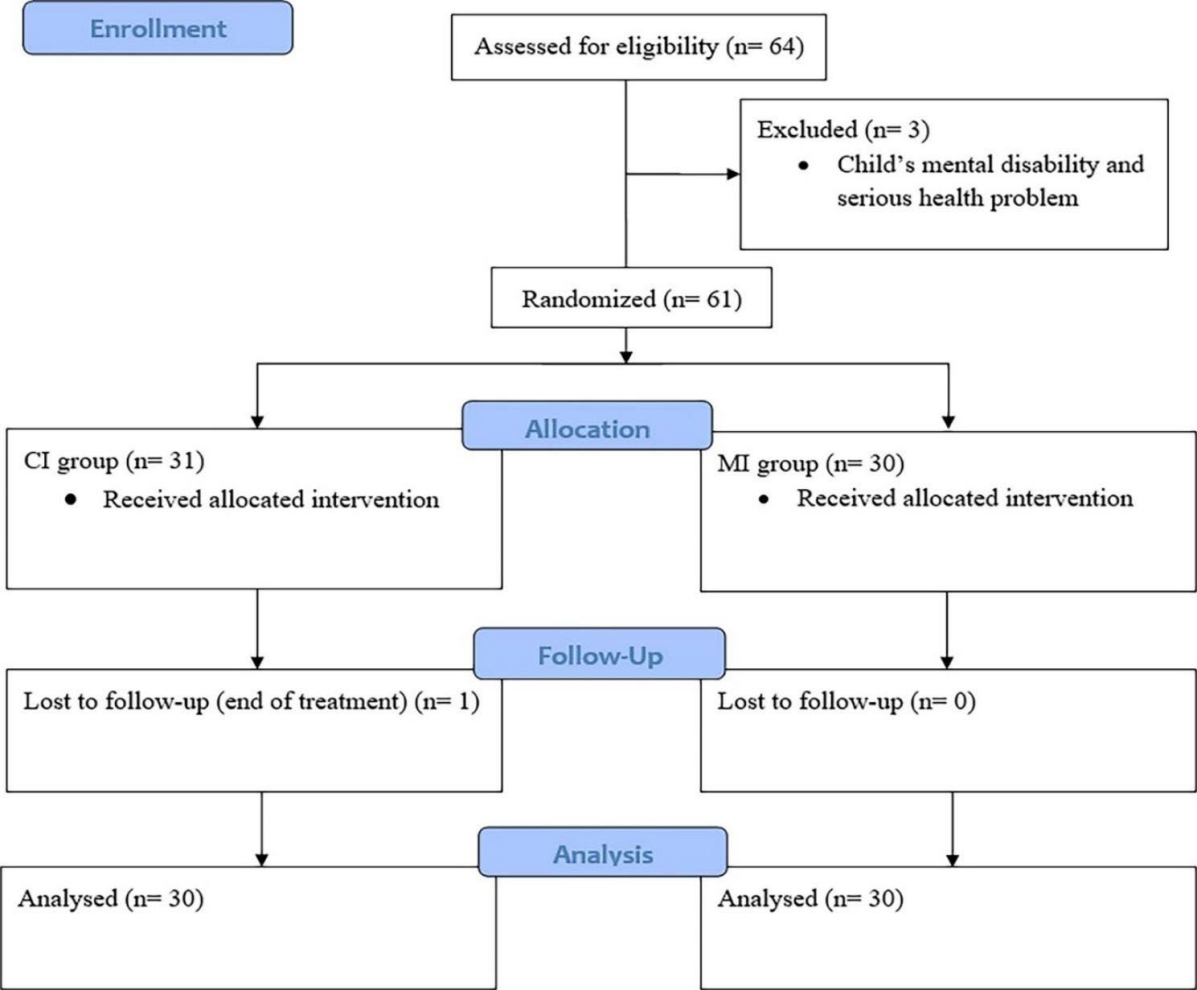
Flow diagram of mothers participating in the study of the effect of motivational interviewing on oral health in leukemic children

This study was conducted on 30 children in the MI group and 31 children in the CI group, as one of the children in the CI group left the hospital before three months follow up as a result of completion of treatment (Fig. 1). The mean children’s ages in the MI group were 4.23 ± 1.41 (ranged from 2 to 6 years old) and 4.32 ± 1.33 (2 to 6 years old) in CI group. There were 16 girls (53.3%) and 14 boys (46.7%) in the MI group, and 15 girls (48.4%) and 16 boys (51.6%) in the CI group. Table 1 shows the demographic characteristics of participants (Table 1).

**Table 1:** Demographic characteristics of Motivational interviewing and Common instruction groups

variable		Motivational interviewing (n = 30)	Common instruction (n = 31)
Academic degree	Uneducated Primary and secondary school High school Diploma Associate degree Bachelor’s degree Master’s degree PhD	6.7% 10.0% 10.0% 43.3% 3.3‌% 20.0% 6.7% 0.0‌%	9.7% 12.9% 16.1% 25.8% 9.7% 19.4% 3.2% 3.2%
Educational level	Pre-high school Diploma Higher education	26.7% 43.3% 30.0%	38.7% 25.8% 35.5%
Economic status	Poor Average Good Excellent	33.3% 50.0% 16.7% 0.0‌%	35.5% 54.8% 9.7% 0.0‌%

Significant differences were found in the amount of children’s plaque index between the MI group and the CI group (0.20 ± 0.04, p-value < 0.001).

In addition, results showed that after the interventions, the mean plaque in the MI group (0.93 ± 0.62, p-value < 0.001) decreased significantly (p-value < 0.001) compared to that of the CI group (1.16 ± 0.79, p-value < 0.001) (Table 2).

**Table 2:** Comparison of baseline (pre-test) and follow-up (post-test) plaque index^*^ of children in MI^**^ and CI^***^ groups

Group	Baseline plaque index	Plaque index after 3 m	Mean difference	95% confidence interval	p-Value
MI	1.16 ± 0.71	0.93 ± 0.62	0.23	0.15 to 0.31	< 0.001
CI	1.21 ± 0.74	1.16 ± 0.79	0.03	-0.2 to 0.7	0.196

^*^ Löe-Silness dental plaque index

^**^ Motivational interviewing

^***^ Common instruction

The results of the paired t-test suggested a significant increase in the mean scores of knowledge, attitude, mother’s self-reported practice concerning child’s oral health, mother’s self-reported practice concerning personal oral health, and motivation in the MI group (p-value < 0.001). Furthermore, in the CI group, no significant changes was observed in mother’s self-reported practice concerning child’s oral health (p-value = 0.573) and motivation (p-value = 0.096); however, a significant difference was observed in mean score of knowledge (p-value < 0.001), attitude (p-value = 0.007), and mother’s self-report practice concerning personal oral health (p-value = 0.050) in the CI group (Table 3).

**Table 3:** Comparison of mean scores of studied variables in Motivational interviewing and Common instruction groups

Variable	Group	Pre-test	Post-test	Mean difference	95% confidence interval	p-Value^*^
Knowledge	MI	58.6 ± 8.71	89.12 ± 6.62	-30.53	-32.80 to -28.25	< 0.001
	CI	53.16 ± 8.22	62.81 ± 9.96	-9.65	-11.72 to -7.58	< 0.001
Attitude	MI	86.39 ± 10.38	95.28 ± 4.48	-8.89	-11.94 to -5.84	< 0.001
	CI	78.06 ± 8/95	79.86 ± 8.48	-1.81	-3.08 to -0.53	0.007
Mother’s self-reported practice concerning child’s oral health	MI	19.05 ± 14.08	41.19 ± 12.40	-22.14	-28.22 to -16.06	< 0.001
	CI	24.52 ± 15.20	26.9 ± 14.73	-2.38	-4.84 to -0.08	0.573
Mother’s self-reported practice concerning personal oral health	MI	53.67 ± 17.9	67.0 ± 17.45	-13.33	-17.86 to -8.80	< 0.001
	CI	46.00 ± 18.86	48.33 ± 19.13	-2.33	-4.67 to 0.00	0.050
Motivation	MI	18.07 ± 1.34	18.93 ± 1.20	-0.87	-1.19 to 0.55	< 0.001
	CI	17.97 ± 1.19	18.13 ± 1.25	-0.17	-0.36 to 0.03	0.096

^*^ Paired t-test

In the MI group, the difference in the changes in the scores of knowledge (22.17 ± 1.49, p-value < 0.001), attitude (10.95 ± 1.23, p-value < 0.001), mother’s self-reported practice concerning child’s oral health (17.42 ± 2.84, p-value < 0.001), mother’s self-reported practice concerning personal oral health (12.09 ± 2.74, p-value < 0.001), and mother’s motivation (0.72 ± 0.17, p-value < 0.001) were significantly greater than those of CI group.

## Discussion

Children’s oral health in high caries risk groups has been ignored and they have encountered some feeding problems [31]. Concurrently, the complications of the therapies used to treat children with leukemia, namely radiotherapy or chemotherapy, have appeared as oral problems and these factors altogether endanger children’s oral health [1]. Therefore, the use of different methods to instruct parents on oral health and the promotion of oral health in these vulnerable groups appear to be necessary. Thus, the present study compared effectiveness of motivational interviewing with a common instruction on mothers’ knowledge, attitude, motivation and practice on the oral health of children with leukemia.

According to the results, the mean score of knowledge in the MI group increased significantly which was consistent with the results of the study by Almomani et al., they examined the effectiveness of motivational interviewing to promote brushing function in people suffering from mental diseases and showed the superiority of the effects of a MI session compared to those of common oral health instructions and announced that MI enhanced the motivation for regular bushing and awareness of the factors threatening oral health while reducing plaque [32]. The study by Yeung et al., also suggested that plaque index, self-regulating behaviors, and individuals’ knowledge of oral health improved after the MI [33] which was consistent with the results of the present study.

According to other results of this study, the mean score of knowledge in the CI group also increased significantly. In the same line, McCann et al., provided children’s oral health instruction using common methods and MI for caregivers and showed that both of these instructions were effective in improving the knowledge required to prevent tooth caries in children [34] and this result was consistent with the results of the present study. It is necessary to point out that the MI method is one of the methods that have been applied recently to encourage people to follow oral health guidelines. Therefore, this instructional method is more effective than the mere provision of information according to the CI methods [35]. On the other hand, parents and in particular mothers are role models who encourage children to learn personal healthcare skills [36]. Furthermore, brushing behavior in parents observed by their children as babies and at a very young age, is often followed by children in their childhood, youth, and even into adulthood [37]. Thus, one of the effective methods of making children get used to brushing is instructing their parents [34].

According to the results of the present study, the mean score of the mother’s attitude toward brushing in the MI group increased significantly. Similarly, Zeidi et al., also indicated a significant improvement in students’ brushing and dental flossing scores after the MI. Overall, we should note that one of the influential factors in promoting oral health behavior is attitude, which has key role in promoting health behaviors has been emphasized in different studies [38–40]. Therefore, we can use MI method to enhance people’s attitudes to promote behaviors connected with oral health.

In this study, the mother’s self-reported practice concerning child’s oral health and personal self-reported oral health improved significantly in MI group. In a similar study by Freudenthal and Bowen, an improvement in the frequency of cleaning teeth and the use of dental floss occurred in parents after conducting MI [41].

Overall, we can significantly help individuals to achieve their goals through the cognitive technique of MI to promote oral health behaviors but it cannot be considered as a method of instruction [42].

Other findings of the current study showed a significant increase in the mean score of mothers’ motivation in MI group. The study by Batliner et al., pointed out the effectiveness of the MI in enhancing motivation in mothers [43]. Overall, the MI led to an increase in the intrinsic motivation in people while also leading to an improvement in mental readiness to make changes to improve and promote health behaviors [44]. In addition, individuals turned from passive participants to active ones leading to an assessment of the costs of changes in behavior and they understand the advantages of improvement in behavior without undergoing any stress which led to an increase in self-efficacy in people. Thus, changes in behavior are facilitated and the increase in mother’s motivation also led to promotion of oral health behavior in this study [38]. Therefore, we recommend using this instructional technique to enhance oral health behavior in mothers of preschoolers.

Furthermore, children’s plaque index decreased significantly in MI method after three months. McCann et al., provided children’s oral health instruction using common methods and MI for caregivers and showed that MI instructional method was more effective in preventing tooth caries in children [34] and this result was consistent with the results of the present study.

Concerning the effectiveness of MI, it is necessary to mention some tips based on prior studies and this research, to consider in future studies: First, short sessions of MI are more effective than long ones [42, 45]; also it is important to include a control group and comparison to examine the effectiveness of this type of instructional method, and also the effectiveness of MI increases at the beginning of follow-ups and then declines over time [46].

In the present study, short instructional sessions of MI were held with mothers as a motivating role model. The changes in children’s performance were evaluated based on mother’s reports all of which were the strengths of this study. Furthermore, the use of clinical examinations to estimate the amount of plaque at the beginning and end of the research was another strength of this study through which we examined the practical results of the provided instructions in changes in plaque as the main cause of tooth caries while also completing the questionnaires. It is necessary to point out that some studies focused their findings on questionnaire results and conducted no clinical examination into the experiences of tooth decay or periodontal diseases in mothers or children. Therefore, it appears necessary to conduct such investigations for future studies. The most frequently referenced measure of supragingival plaque is the Silness-Löe Plaque Index or Pl, which uses an ordinal scale of 0 to 3 per single teeth and a total measure of mean plaque index per child. Therefore, in this article, the Silness-Löe plaque index has been used for measuring the short term clinical outcome of the interventions which revealed less than 20% reduction in plaque index of MI group when compared with the baseline value as a scale. Considering the clinical relevance of such difference between the two intervention groups, we recommend increasing the length of follow-up to at least six months in future studies to measure the ultimate effects of the interventions and to add long term clinical outcomes such as tooth decay. In this study, the principal investigator (NF) performed interventions for both CI and MI groups which could have worked as a potential source of bias but as examinations were performed by a dentist of Mahak institute, we believe the bias might be minimum. In addition, we suggest investigating confounding variables like simultaneous instructions from other resources like mass media or visiting dentists be considered in future studies. The other noteworthy point is that the findings are not generalizable, thus, we suggest instructing all young age groups and children with different socioeconomic conditions and their parents by using MI methods to assess oral health in future studies.

## Conclusion

According to the findings, instruction using motivational interviewing revealed to be effective in improving oral health and reducing plaque index in children with leukemia and it is recommended to promote the oral health of leukemic children with participation of their mothers in places that are constantly present for treatment.

## Electronic supplementary material

Below is the link to the electronic supplementary material.

Supplementary Material 1

## Data Availability

The datasets generated and/or analyzed during the current study are not publicly available considering that we have not required consents to publish this data, but are available from the corresponding author on reasonable request.

## List of abbreviations

MI: Motivational interviewing
CI: Common instruction
PI: Plaque index
