# Supplementary material for: The effectiveness of motivational interviewing on the oral health of leukemic children and oral health care knowledge, attitude and practice of their mothers: a hospital-based intervention
Source: BMC Pediatr. 2023 May 24;23:261. doi: 10.1186/s12887-023-04078-y (PMC10207771; doi:10.1186/s12887-023-04078-y)
Supplement: Supplementary file 1 — Supplementary Material 1 [file 12887_2023_4078_MOESM1_ESM.pdf]

Questionnaire about mother's oral-health knowledge, mother's practice about children, demographic data and child's clinical examination:

Knowledge questions:

1.1. The first primary tooth erupts averagely at 6 months of age.

Yes ☐ No ☐ I do not know ☐

1.2. Bacteria responsible for dental caries often transfer from mother to child.

Yes ☐ No ☐ I do not know ☐

1.3. Fluoridated toothpastes should not be used for children under 3 years of age.

Yes ☐ No ☐ I do not know ☐

1.4. White lines or spots are the first signs of caries in children.

Yes ☐ No ☐ I do not know ☐

1.5. Tooth cleaning and tooth brushing should be started following the eruption of the first primary tooth.

Yes ☐ No ☐ I do not know ☐

1.6. Fluoridated toothpastes prevent dental caries.

Yes ☐ No ☐ I do not know ☐

1.7. Children should not use fluoride.

Yes ☐ No ☐ I do not know ☐

1.8. Frequency of intake of sugary substances plays a more important role than the total amount of consumption of sugar in caries development.

Yes ☐ No ☐ I do not know ☐

1.9. Irrigation of teeth with copious water after tooth brushing can effectively prevent caries.

Yes ☐ No ☐ I do not know ☐

1.10. Consumption of xylitol in the form of chewing gum or snacks can decrease dental caries.

Yes ☐ No ☐ I do not know ☐

1.11. Bottle-feeding can cause early childhood caries..

Yes ☐ No ☐ I do not know ☐

1.12. Carious primary teeth need to be restored.

Yes ☐ No ☐ I do not know ☐

1.13 Children with systematic diseases need to receive fluoride supplements.

Yes ☐ No ☐ I do not know ☐

1.14 Children with systematic diseases should visit the dentist at least twice a year.

Yes ☐ No ☐ I do not know ☐

The following items can cause dental caries.

Item	Yes	No	I do not know
1.15 Candy			
1.16 Fruit juice			
1.17 Cola			
1.18 Cheese			
1.19 Tea			

Attitude questions:

2.1. It is important for parents to clean children's teeth.

Strongly agree ☐ Agree ☐ Neither agree nor disagree ☐

Disagree ☐ Strongly disagree ☐

2.2.Primary teeth do not need much care because they eventually fall anyway.

Strongly agree ☐ Agree ☐ Neither agree nor disagree ☐

Disagree ☐ Strongly disagree ☐

2.3.I believe that dental problems can lead to child's general health problems.

Strongly agree ☐ Agree ☐ Neither agree nor disagree ☐

Disagree ☐ Strongly disagree ☐

2.4.Tooth decay in children is preventable.

Strongly agree ☐ Agree ☐ Neither agree nor disagree ☐

Disagree ☐ Strongly disagree ☐

2.5.Regular dental visits are important to prevent oral diseases.

Strongly agree ☐ Agree ☐ Neither agree nor disagree ☐

Disagree ☐ Strongly disagree ☐

2.6.Children with systematic diseases have a higher risk for tooth decay and oral diseases.

Strongly agree ☐ Agree ☐ Neither agree nor disagree ☐

Disagree ☐ Strongly disagree ☐

Mother's practice concerning child’s oral health questions:

3.1. Who brushes the teeth of your child?

- ☐ He/she does not brush yet
- ☐ Himself/ Herself
- ☐ My other child
- ☐ Himself/ Herself under the supervision of adults
- ☐ Adults

3.2.How often does your child brush his/her teeth?

- ☐ Irregularly or never
- ☐ Once a week
- ☐ Several times (2-3 times) a week
- ☐ Once a day
- ☐ More than once a day

3.3.How often do you take your child to the dentist?

- ☐ When a problem occurs
- ☐ Once every six months to a year
- ☐ Irregularly
- ☐ etc.

3.4. Does your child rinse his/her mouth after eating sugary foods?

- ☐ Always
- ☐ Most of the time
- ☐ Occasionally
- ☐ Never

3.5. How often does your child have a sweet snack or sweet drink (warm or cold)?

- ☐ Three times or more per day

☐ Around 2 times per day

☐ Once a day

☐ Occasionally, not everyday

☐ Rarely

3.6. Do you use fluoridated toothpaste when brushing your child's teeth?

☐ Always

☐ Most of the time

☐ Occasionally

☐ Never

☐ I do not know

3.7. Does the child sleep with a bottle (other than a glass containing water) or a breast in the mouth during sleep?

☐ With breast

☐ With bottle

☐ With both

☐ Niether

3.8. If the child wakes up in the middle of the night, what do you usually do to make her/ him sleep?

☐ Breast feeding

☐ Milk bottle

☐ A sweet liquid bottle

☐ Water bottle

☐ Without feeding

☐ Other behaviors such as calming her/ him down and walking or...

3.9. How many times a year does your child use fluoride supplements such as fluoride varnish?

- ☐ More than 4 times
- ☐ 4 times
- ☐ 3 times
- ☐ Less than twice
- ☐ Does not use

Mother's practice concerning personal oral health questions:

4.1. How often do you brush your teeth?

- ☐ Irregularly or never
- ☐ Once a week
- ☐ Several times (2-3 times) a week
- ☐ Once a day
- ☐ More than once a day

4.2. How often do you use dental floss?

- ☐ Never
- ☐ Occasionally
- ☐ Several times (2-3 times) a week
- ☐ Once a day
- ☐ More than once a day

4.3. Do you use fluoridated toothpaste when brushing your teeth?

- ☐ Always
- ☐ Most of the time

- [ ] Occasionally
- [ ] Never

4.4.How often do you have a sweet snack or sweet drink (warm or cold)?

- [ ] Three times or more per day
- [ ] Around 2 times per day
- [ ] Once a day
- [ ] Occasionally, not everyday
- [ ] Rarely

4.5.When was your last dental visit?

- [ ] Over the past six months
- [ ] Six months to a year ago
- [ ] A year or two ago
- [ ] Two to five years ago
- [ ] More than five years ago
- [ ] Never
- [ ] I do not remember

Motivation questions:

Answer on a scale of 0 to 10:

1. How interested are you that your child does not have dental problems?
2. How confident are you that you can improve your child's oral health?

Demographic questions:

Phone number:

Gender of the child?

Boy ☐ Girl ☐

Age of the child?

Level of education of mother?

☐ Illiterate

☐ Below high school diploma

☐ High school

☐ High school diploma

☐ College degree

☐ Bachelor’s degree

☐ Master’s degree

☐ Doctorate degree

Socioeconomic status of the family?

☐ Poor

☐ Moderate

☐ Good

☐ Excellent

Oral examination:

Scoring of PI was as follow:

- Score of 0: absence of dental plaque;
- Score 1: presence of plaque in gingival margin which cannot be seen with the unaided eye;
- Score 2: a moderate amount of soft deposits in the gingival margin that can be seen with the unaided eye;
- Score 3: the presence of soft materials in the gingival margin or tooth and gingival edge.

The mean plaque of all teeth for each child was sum up as well (ranging from 0 to 3).

<table>
<tr><td>E</td><td>B</td><td rowspan="2">maxilla</td><td>D</td><td></td></tr>
<tr><td></td><td></td><td></td><td></td></tr>
<tr><td></td><td>D</td><td rowspan="2">mandible</td><td>B</td><td>E</td></tr>
<tr><td></td><td></td><td></td><td></td></tr>
</table>
